# Treatment of central serous chorioretinopathy: new options for an old disease

**DOI:** 10.1038/s41433-025-03894-z

**Published:** 2025-07-04

**Authors:** Yoon Jeon Kim, Sobha Sivaprasad, Tariq Aslam, Polona Jaki Mekjavić, Vilma Jūratė Balčiūnienė, Linda Visser, Antonia M. Joussen, Young Hee Yoon, Timothy Y. Y. Lai, Annabelle A. Okada

**Affiliations:** 1 Department of Ophthalmology, Asan Medical Center, University of Ulsan College of Medicine, Seoul, South Korea; 2 UCL Institute of Ophthalmology, London, UK; 3 Moorfields NIHR Biomedical Research Centre, Moorfields Eye Hospital, London, UK; 4 Division of Pharmacy and Optometry, Faculty of Biology, Medicine and Health, The University of Manchester, Manchester, UK; 5 Manchester Royal Eye Hospital, Manchester, UK; 6 Eye Hospital, University Medical Centre Ljubljana, Ljubljana, Slovenia; 7 Faculty of Medicine, University of Ljubljana, Ljubljana, Slovenia; 8 Department of Ophthalmology, Lithuanian University of Health Sciences, Kaunas, Lithuania; 9 Division of Ophthalmology, Stellenbosch University, Cape Town, South Africa; 10 Tygerberg Hospital, Cape Town, South Africa; 11 Charité – University Medicine Berlin, Berlin, Germany; 12 Department of Ophthalmology & Visual Sciences, The Chinese University of Hong Kong, Hong Kong, China; 13 Department of Ophthalmology, Kyorin University School of Medicine, Tokyo, Japan

**Keywords:** Retinal diseases, Scientific community

## Abstract

Central serous chorioretinopathy (CSC) is a common ocular disease that causes vision loss, particularly in people of working age. Although it was first described over a century ago, research has expanded in recent years, transforming the understanding and management of this complex condition. Here, we propose clinical recommendations for the treatment and management of CSC, based on evidence from the published literature and the consensus of an international group of retinal experts. Recent evidence describing the pathogenesis of and risk factors for CSC focuses on possible dysfunction of the choroid and retinal pigment epithelium, and the role of corticosteroids. It is suggested that CSC lies within the spectrum of pachychoroid disorders that share the characteristic of thickened choroidal tissue. Available evidence supports half-dose (or half-fluence) verteporfin photodynamic therapy as the treatment of choice for CSC to reduce choroidal hyperpermeability. A subset of patients with chronic CSC can develop choroidal neovascularisation, and these patients should be treated with intravitreal anti-vascular endothelial growth factor agents. Finally, posterior multifocal pigment epitheliopathy is a recognised variant of CSC that may progress to large areas of exudative retinal detachment. We propose a step-wise chart for clinical decision-making in the management and treatment of CSC. New data on long-term visual outcomes and the pathogenesis of CSC in relation to the pachychoroid disease spectrum provide a better understanding to inform our management of this disease.

## Introduction

Central serous chorioretinopathy (CSC) is a common chorioretinal disease that causes vision loss. It predominantly affects people of working age and is six times more common in men than women [1]. Reported cases of CSC involving both eyes are as high as 40%, and the rate of bilateral involvement at the time of initial diagnosis is around 4% [2]. Recent developments in genetics and ocular imaging have improved understanding of the pathophysiology of CSC, and the evidence base is growing following publication of the results of randomised controlled trials (e.g., PLACE [Half-Dose Photodynamic Therapy versus High-Density Sub-threshold Micropulse Laser Treatment in Patients with Chronic Central Serous Chorioretinopathy trial]) and large, retrospective, non-randomised treatment studies, from which optimal treatment guidelines can be developed [3, 4].

In this article, we provide an overview of the risk factors and pathogenesis of CSC. We also summarise published evidence for the treatment of CSC and propose an evidence-based flowchart containing recommendations for practical disease management and the treatment of phenotypic variations.

## Methods

This article is based on a review of the literature and a consensus among retinal experts from the Vision Academy. The Vision Academy is a group of over 80 international experts who, through their collective expertise, provide consensus guidance for managing clinically challenging situations, especially in areas of controversy or with insufficient conclusive evidence (www.visionacademy.org). The Vision Academy is sponsored by Bayer.

A literature search was performed using the PubMed database to identify relevant publications. Keywords for the search were “central serous chorioretinopathy” or “central serous retinopathy”, plus keywords grouped into the following categories:

1. Pathophysiology: pachychoroid spectrum diseases (“pachychoroid” or “choroidal thickness”), genetic risk factors (“genetics” or “risk factors”), drug-induced CSC or CSC-like disease (“corticosteroid” or “mineralocorticoid”)
2. Treatment: “laser”, “anti-vascular endothelial growth factor”, “aflibercept”, “ranibizumab”, “bevacizumab”, “brolucizumab”, “photodynamic therapy”, “mineralocorticoid antagonist”, “treatment”, “therapy”
3. Testing: “visual acuity”, “visual function”, “visual outcome”, “microperimetry”, “fundus autofluorescence”, “angiography”, “optical coherence tomography”
4. Complications or variant forms of CSC: “choroidal neovascularisation”, “retinal pigment epithelium” and “atrophy”, “bullous or serous retinal detachment”, “multifocal posterior pigment epitheliopathy”

In total, 953 relevant papers (446 articles regarding pathophysiology, 838 articles discussing treatment and 308 articles describing complications) published from 2015 to 2020 were identified, and 72 key articles presenting important features of CSC were critically reviewed for this manuscript. Additionally, several papers published in 2021 and 2022 were included following discussion among the authors. The information gathered from the literature was combined with the clinical experiences of the authors to inform the development of recommendations for the management of CSC. These recommendations were subsequently reviewed, commented upon and endorsed by a majority of the Vision Academy’s membership before finalisation. Vision Academy members were asked to rate their agreement with the proposed recommendations using the options “strongly agree”, “agree”, “neither agree nor disagree”, “disagree” and “strongly disagree”. More than 50% of members were required to respond to the survey for its results to be considered valid. Respondents were also asked for the reimbursement status of treatment in their country of practice (reimbursed, out-of-pocket or a combination of the two), to determine whether this may have influenced their responses. Biases were assessed using χ^2^. Endorsement was established if 50% or more of respondents indicated that they agreed or strongly agreed with a recommendation; consensus was considered “strong” if more than 75% of respondents agreed or strongly agreed. The list of Vision Academy members who contributed to the recommendations is provided at the end of this paper.

## Pathogenesis of CSC

### Choroidal dysfunction

Whether the primary pathogenic lesion of CSC is within the choroid or the retinal pigment epithelium (RPE) or a more systemic abnormality is still controversial. However, increased choroidal thickness and choroidal vessel dilation are widely accepted as being characteristic of CSC.

It is now thought that CSC arises as a result of abnormal choroidal blood flow regulation, leading to ischaemia at the level of the choriocapillaris [5]. Ischaemia at the level of the choriocapillaris results in focal or diffuse dysfunction of the RPE and subsequent accumulation of subretinal fluid (SRF) [4]. Unrelenting oxidative stress in the ischaemic region is thought to be associated with a delay in arterial filling and chronic venous congestion, resulting in the production of pro-inflammatory factors and hyperpermeability. This causes the choroid to thicken and can lead to subretinal material deposition [4].

Studies have shown that patients with chronic CSC exhibit a higher choroidal vascularity index [6, 7] and a reduced stromal area to choroidal area ratio [8], as well as enlarged choroidal vessels [9], compared with control individuals. A meta-analysis including 397 eyes with CSC and 228 unaffected fellow eyes revealed that many of the choroidal changes seen in affected eyes were also present in the contralateral eyes, suggesting a more widespread choroidal dysfunction [10]. In this context, recent studies indicate a relationship between CSC and pachychoroid spectrum diseases, in which choroidal thickening plays a key pathogenic role [11]. Increased choroidal thickness is a primary feature of pachychoroid disease, and intervortex venous anastomoses have recently been identified in CSC [12]. Spaide et al. suggested venous overload choroidopathy as the unifying feature of CSC-related diseases [13].

### RPE dysfunction

The role of the RPE in the pathogenesis of CSC is not well understood. It is generally believed that fluid leakage at the level of the RPE into the subretinal space is increased due to microcirculation abnormalities in choroidal capillaries [14]. It has been hypothesised that damaged RPE cells in areas of leakage overburden the metabolically functioning RPE, resulting in the persistence of serous fluid [4, 15]. In cases of continuous insufficient microcirculation, disease recurrence and permanent tissue damage occur. It has been suggested that damage to the RPE may result in the secondary formation of anti-retinal antibodies that may affect the clinical course of CSC [16].

### The corticosteroid hypothesis

A study in rats found that choroidal vasodilation and vessel hyperpermeability occurred following intravitreal injection of aldosterone, with elongation of RPE microvilli and increased choroidal thickness similar to observations in CSC [17]. In addition, a comparative analysis of SRF samples from patients with CSC versus control individuals with rhegmatogenous retinal detachment suggested dysregulation of the alternative complement pathway and the glucocorticoid and mineralocorticoid systems (abnormal levels of aldosterone, angiotensin and corticosteroid-binding globulin) [18].

### Risk factors

Several systemic risk factors are associated with the development of CSC (Table 1). Patients receiving corticosteroid therapy are at high risk of developing the disease, with systemic (oral or intravenous) corticosteroid use recognised as an independent risk factor [19]. CSC has also been described following local administration of corticosteroids via inhaled, intranasal, epidural, intra-articular, topical/dermal and periocular routes [19]. Steroid-induced CSC is potentially an idiosyncratic response in vulnerable individuals, with less male predilection than is observed with non-steroid-induced disease, frequent bilaterality and commonly atypical presentation [20]. This response is not limited to exogenous corticosteroid use; it also occurs due to excess endogenous corticosteroid production resulting from pituitary gland tumours, adrenal gland disease, ectopic adrenocorticotropic hormone-secreting tumours and familial Cushing syndrome [4, 21]. Additionally, CSC is the leading cause of acquired retinal/choroidal visual impairment during pregnancy [22]. Endogenous corticosteroid levels increase during pregnancy, and changes in progesterone and testosterone levels, the renin–angiotensin system and blood volume occur [4].

**Table 1:** Risk factors for CSC.

Category	Risk factors	Key considerations
Corticosteroid use	Systemic corticosteroid use (oral, intravenous), local corticosteroid administration (inhaled, intranasal, epidural, intra-articular, topical, periocular) [19]	Steroid-induced CSC shows frequent bilaterality, atypical presentation and less male predilection than non-steroid-induced CSC [19]
Endogenous hormonal changes	Endogenous hypercortisolism (Cushing syndrome, adrenal/pituitary tumours, ectopic adrenocorticotropic hormone-secreting tumours) [4]; pregnancy-related hormonal changes (elevated cortisol, progesterone, testosterone) [22], renin–angiotensin system activation, increased blood volume [4]	CSC is the leading cause of pregnancy-related acquired retinal/choroidal visual impairment [22]
Autonomic dysfunction and sleep disorders	Sympathetic overactivation [23], decreased parasympathetic activity [98], obstructive sleep apnoea (5× increased risk) [23]	Association between CSC and autonomic imbalance [23]; sleep apnoea screening is recommended in patients with CSC [99]
Infectious and drug-related factors	*Helicobacter pylori* infection, sympathomimetic agents (pseudoephedrine, oxymetazoline), MMDA [25], phosphodiesterase-5 inhibitors (sildenafil, tadalafil) [26]	Pathophysiological mechanisms of *H. pylori* in CSC remain unclear [24]; drug-induced CSC should be considered in at-risk patients [3, 36]
Psychological and personality factors	Psychological stress, Type A personality [23], antipsychotic medication use, depression, adjustment disorder [28]	Recent studies using validated questionnaires found no clear association between CSC and maladaptive personality traits [29]
Genetic risk factors	Single-nucleotide polymorphisms in *CFH* (rs3753394, rs1329428, rs800292) [30, 31], *VIPR2* (rs3793217) [32], *CDH5* [4], *TNFRSF10A-LOC389641*, *GATA5* [33]	Genetic variations may influence choroidal circulation and steroid response, but functional impacts require further research [31]

*CFH* complement factor H, *CSC* central serous chorioretinopathy, *MMDA* 3-methoxy-4,5-methylenedioxyamphetamine.

Additional CSC risk factors include sympathetic overactivation and decreased parasympathetic activity and obstructive sleep apnoea, with a population-based study showing that patients with CSC are five times more likely to have obstructive sleep apnoea than age- and sex-matched controls [23]. Hyperopia is also associated with an increased risk of CSC, while myopia is associated with a decreased risk [23]. Several previous studies reported an association between *Helicobacter pylori* infection and CSC, but the potential pathophysiology behind this is unknown [24]. Use of sympathomimetic agents such as pseudoephedrine and oxymetazoline nasal sprays, as well as 3-methoxy-4,5-methylenedioxyamphetamine (MMDA), has been associated with CSC [25], and phosphodiesterase-5 inhibitors (sildenafil and tadalafil) are also potentially associated [26].

Chatziralli et al. investigated any potential association between CSC and a range of patient characteristics, including stress and Type A personality [23]. Continuous stress would be expected to lead to elevated cortisol levels in individuals with a Type A personality [4]. Antipsychotic medication use, psychological stress, adjustment disorder and depression have also been associated with an increased risk of CSC recurrence [27, 28]. However, recent studies using validated questionnaires have shown no link between maladaptive personality traits and CSC [29].

Both genetic risk and protective factors have been identified in patients with CSC, but their precise role in the disease pathogenesis remains unclear [30]. The complement factor H (*CFH*) gene encodes for the protein factor H, known as adrenomedullin-binding protein-1, which binds to adrenomedullin to stimulate choroidal dilation [30]. Several candidate-gene studies have identified single-nucleotide polymorphisms in *CFH*. Specifically, rs3753394 is located in the region of the *CFH* promoter, between a glucocorticoid response element and a possible histone H4 gene-binding site, H4TF-1, that may be a common regulatory element and hence could influence gene expression [31]. Additionally, the rs1329428 polymorphism is known to bind to transcriptional regulatory proteins, which, in turn, may have downstream consequences [31]. Further information about the functional repercussions of identified single-nucleotide polymorphisms is required to elucidate the role of *CFH* in CSC. Variants in the *CFH* gene (rs800292) and the *VIPR2* gene (rs3793217) have been found to be significantly associated with both pachychoroid disease and CSC [32]. In addition, *CDH5* has been linked to increased susceptibility to CSC [4]. Other notable candidate genes in CSC are rs13278062 at *TNFRSF10A-LOC389641* and rs6061548 near *GATA5* [33].

### Clinical characteristics of CSC

CSC overlaps with other disorders within the pachychoroid spectrum [3, 11]; a retrospective study including 60 patients with unilateral active CSC found choroidal hyperpermeability to be present in 93% of participants [34]. There is currently no widely accepted classification system for CSC. Although several classifications have been proposed, the diverse clinical features and disease course are still debated. Many researchers make a distinction between acute and chronic CSC, based on the duration of SRF [3, 4]. Regardless, multimodal imaging is essential to evaluate the disease precisely. By using fluorescein angiography (FA), indocyanine green angiography (ICGA), optical coherence tomography (OCT) and fundus autofluorescence, clinicians can differentiate acute CSC from chronic CSC and make a differential diagnosis from other overlapping conditions [1, 3, 4].

**Fig. 1:**
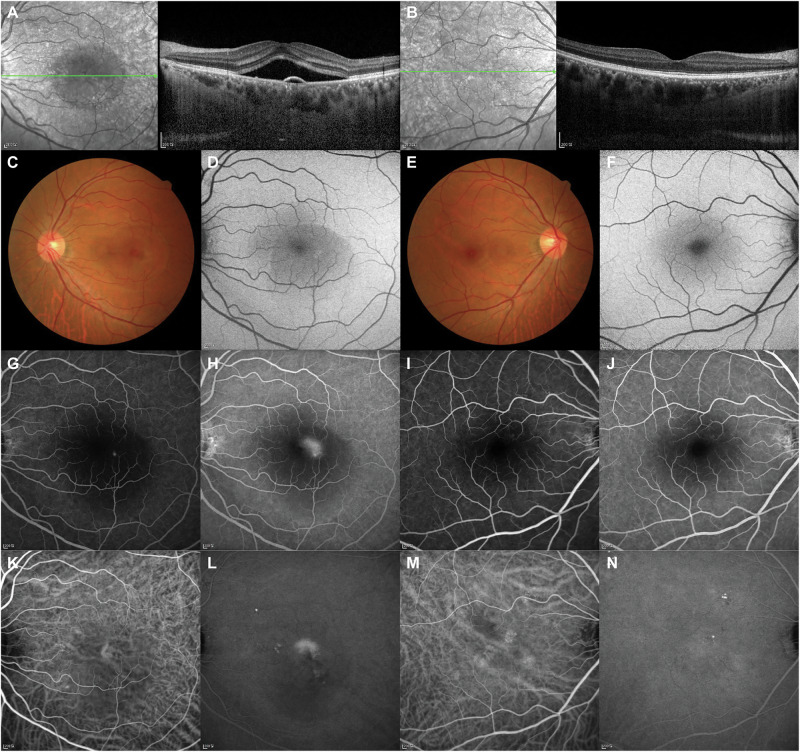
Multimodal imaging of a 45-year-old male patient diagnosed with acute CSC in the left eye. SRF involving the fovea and the thick choroid are detected on OCT (**A**). Thick choroid in the fellow eye is also noted on OCT (**B**). Serous macular elevation seen on colour fundus photo (**C**) shows a hypo-autofluorescent area and hyperautofluorescent border on fundus autofluorescence (**D**), compared with the fellow eye (**E**, **F**). On FA, there is a focal area of hyperfluorescent dye leakage, which increases and ascends in the subretinal space to produce a “smoke stack” leakage pattern (**G**, **H**), compared with that of the fellow eye (**I**, **J**). On ICGA, hyperfluorescent areas of choroidal vascular hyperpermeability are noted (**K**, **L**), and similar patterns are also seen in the fellow eye (**M**, **N**). CSC central serous chorioretinopathy, FA fluorescein angiography, ICGA indocyanine green angiography, OCT optical coherence tomography, SRF subretinal fluid.

Acute CSC (Fig. 1) is characterised by acute-onset accumulation of SRF (of 3–4 months’ duration) and/or pigment epithelial detachment (PED) with a good visual prognosis [1], and has been shown to resolve spontaneously in the majority of cases [3, 4].

**Fig. 2:**
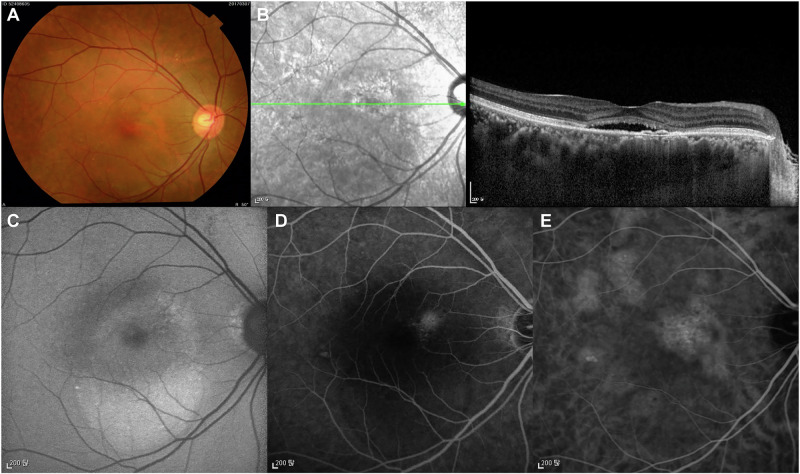
Multimodal imaging of a 54-year-old male patient with chronic CSC. Colour fundus photography shows macular serous elevation and pigmentary abnormalities, some of which correspond to the areas of leakage seen on FA (**A**). OCT demonstrates SRF, flat irregular PED and photoreceptor outer segment atrophy. Diffuse severe choroidal thickening with dilated veins in Haller’s layer and choriocapillaris attenuation below the flat irregular PED are present (**B**). Fundus autofluorescence shows mostly hyperautofluorescent abnormalities, “fluid track”, associated with RPE atrophy induced by the chronic presence of SRF (**C**). Focal areas of hyperfluorescent leakage are seen on FA (**D**). Dilated choroidal vessels and choroidal vascular hyperpermeability are evident on ICGA (**E**). CSC central serous chorioretinopathy, FA fluorescein angiography, ICGA indocyanine green angiography, OCT optical coherence tomography, PED pigment epithelial detachment, RPE retinal pigment epithelial, SRF subretinal fluid.

Chronic CSC (Fig. 2) is characterised by SRF with various levels of PED and/or RPE decompensation secondary to choroidal abnormalities [1, 3, 4]. Patients with chronic CSC typically have persistent SRF for longer than 4–6 months [1]. The typical feature of the RPE atrophic tract in the fundus is located inferiorly due to the gravitational effect of long-standing SRF [4]. Widespread RPE decompensation with changes on fundus autofluorescence is a characteristic finding [4]. Several risk factors for prolonged CSC duration that are present at initial diagnosis have been evaluated, including subfoveal choroidal thickness over 500 µm, PED height above 50 µm and age over 40 years [35]. Recurrences are common, particularly in cases with no intervention, and range from 15% to 50% depending on the study design and follow-up periods [36].

**Fig. 3:**
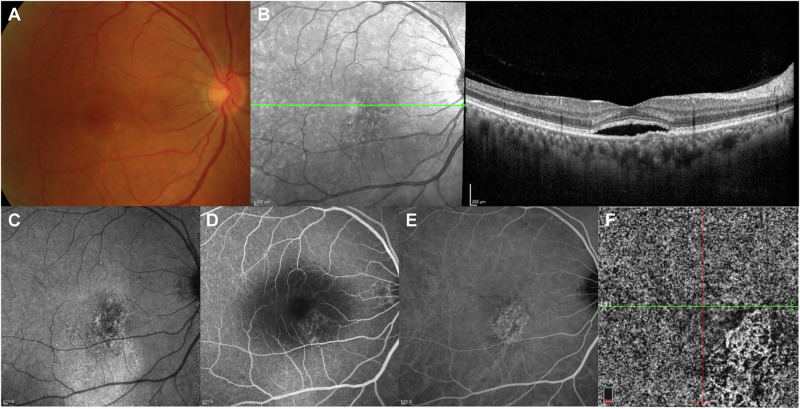
Multimodal imaging of a 57-year-old male patient with chronic CSC complicated by type 1 CNV. Retinal pigmentary changes on fundus photography (**A**), accompanied by foveal SRF and a flat irregular PED, are observed on OCT (**B**). Fundus autofluorescence shows mostly hyperautofluorescent abnormalities, “fluid track”, representing RPE atrophy induced by chronic presence of SRF (**C**). Leakage of fluorescein and diffuse RPE alterations are present on FA (**D**). ICGA shows a certain degree of demarcation that is suggestive of type 1 CNV (**E**). OCT-A clearly shows the neovascular network (**F**). CNV choroidal neovascularisation, CSC central serous chorioretinopathy, FA fluorescein angiography, ICGA indocyanine green angiography, OCT optical coherence tomography, OCT-A optical coherence tomography angiography, PED pigment epithelial detachment, RPE retinal pigment epithelial, SRF subretinal fluid.

A subset of patients with chronic CSC can develop choroidal neovascularisation (CNV), primarily type 1 macular neovascularisation [37] (Fig. 3). There seems to be a clinical overlap between CSC with CNV and pachychoroid neovasculopathy, and recent studies have suggested that both diseases share the same pathogenesis and ultimately may be the same disease [38]. A retrospective case series revealed that CNV was present in the clinical course of 2–4% of chronic CSC cases [36]. A study of chronic CSC with type 1 CNV found that these eyes often exhibit a flat, irregular PED [39]; CNV has been found to be significantly associated with chronic CSC, choroidal vascular hyperpermeability and choriocapillary hypoperfusion [40]. Older age, hypertension, pigmentary changes and double-layer sign have also been suggested as possible risk factors for type 1 CNV secondary to CSC [40, 41].

**Fig. 4:**
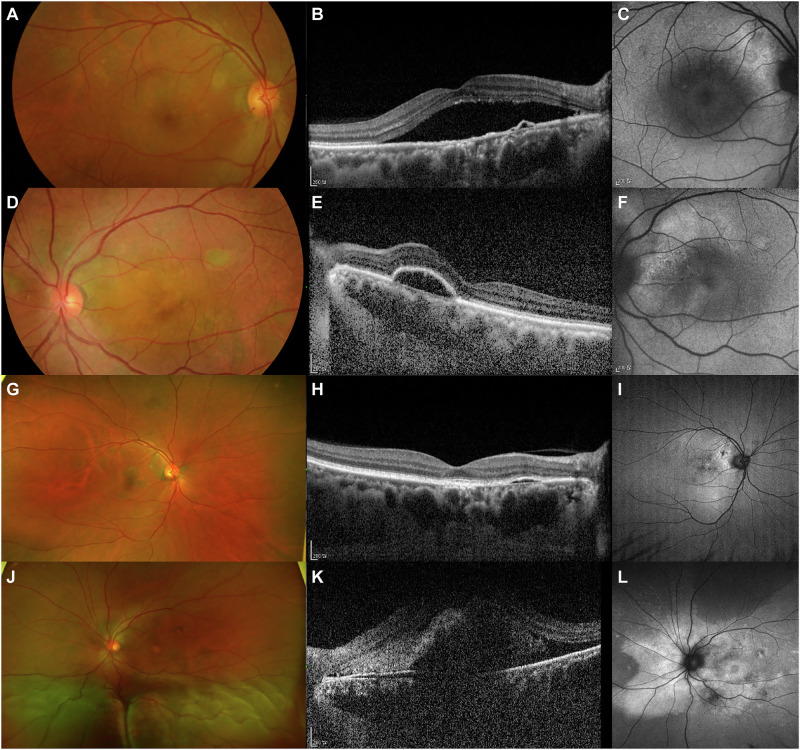
Multimodal imaging of a 56-year-old male patient with bullous CSC. At initial presentation, the right eye shows macular serous elevation on colour fundus photography (**A**) and SRF involving the fovea and focal PED on OCT (**B**), which corresponds to a hypo-autofluorescent area on FAF (**C**). The left eye shows RPE alterations on colour fundus photography (**D**) and large dome-shaped PED on OCT (**E**), corresponding to a hyperautofluorescent area on FAF (**F**). One year after focal laser treatment in the right eye, macular pigmentary changes are visible on wide fundus photography (**G**), with complete resolution of SRF on OCT (**H**). Wide FAF shows mostly hyperautofluorescent changes due to RPE stress induced by chronic presence of SRF and perifoveal hypofluorescent spots due to laser scars and RPE clumping (**I**). Meanwhile, the left eye developed bullous CSC. Wide fundus photography shows the bullous elevated retina inferiorly (**J**). OCT image demonstrates a moderate amount of turbid subfoveal SRF and large PED (**K**). Wide FAF shows mostly hyperautofluorescent changes reaching the peripheral lesion (**L**). CSC central serous chorioretinopathy, FAF fundus fluorescein angiography, OCT optical coherence tomography, PED pigment epithelial detachment, RPE retinal pigment epithelial, SRF subretinal fluid.

Multifocal posterior pigment epitheliopathy associated with exudative retinal detachment in CSC, also known as bullous CSC, is a rare variant of the disease that is characterised by severe serous retinal detachment, particularly in the inferior quadrants [4] (Fig. 4). RPE tears are very common in patients with this form of CSC, who typically present with PED, often located in the posterior pole, with internal hyperreflectivity representing turbid fibrin [4]. Kawamura and colleagues described eight patients who had CSC with bullous retinal detachment, several diffuse leakage spots and/or fibrin formation [42].

### Differential diagnosis of CSC

CSC should be differentiated from various pachychoroid-related diseases, neovascular conditions, inflammatory disorders, tumours, systemic diseases, genetic disorders and drug-induced retinopathy, all of which can present with serous retinal detachment or SRF accumulation (Table 2).

**Table 2:** Differential diagnosis of CSC.

Category	Conditions	Key differentiation
Pachychoroid-associated diseases	Pachychoroid pigment epitheliopathy, peripapillary pachychoroid syndrome, PCV, CNV with increased choroidal thickness, focal choroidal excavation [45], peripheral exudative haemorrhagic chorioretinopathy [44]	PCV shows a branching neovascular network on ICGA; CSC exhibits focal leakage without neovascularisation [44]
Neovascular diseases	AMD with type 1 macular neovascularisation [46]	AMD and PCV show persistent leakage on FA [41]
Inflammatory diseases	VKH disease [50], posterior scleritis [51]	VKH disease is always bilateral, and patients may recall systemic prodromal symptoms. In the acute phase, the choroid is diffusely and massively thickened to an extent not seen with CSC, and anterior chamber inflammatory cells may be observed. VKH disease presenting in the chronic phase may show severe anterior segment inflammation, with posterior iris synechia and vitritis [50] Posterior scleritis can be distinguished by B-mode ultrasonographic findings [51]
Choroidal tumours and systemic malignancies	Choroidal melanoma [53], choroidal haemangioma [54], metastases, leukaemia infiltration, Waldenström’s macroglobulinaemia, choroidal lymphoma, bilateral diffuse uveal melanocytic proliferation, paraneoplastic vitelliform maculopathy [46]	Tumours and haematological malignancies can be identified using ultrasound [54], angiography, computed tomography/magnetic resonance imaging and systemic evaluations [46]
Genetic and developmental disorders	Best vitelliform macular dystrophy, RP1L1-associated occult macular dystrophy, central areolar choroidal dystrophy, optic pit maculopathy, uveal effusion syndrome, tilted disc syndrome, dome-shaped macula [46]	Genetic disorders show family history, absence of leakage on FA, and distinct patterns on OCT; developmental anomalies present with structural variations on OCT and ultrasound [46]
Drug-induced serous retinopathy	MAPK inhibitor-associated retinopathy [55], FGFR inhibitor-related CSC [56]	History of systemic therapy with MAPK or FGFR inhibitors and the absence of choroidal thickening help differentiate drug-induced cases from CSC [55, 56]

*AMD* age-related macular degeneration, *CNV* choroidal neovascularisation, *CSC* central serous chorioretinopathy, *FA* fluorescein angiography, *FGFR* fibroblast growth factor receptor, *ICGA* indocyanine green angiography, *MAPK* mitogen-activated protein kinase, *OCT* optical coherence tomography, *PCV* polypoidal choroidal vasculopathy, *VKH* Vogt–Koyanagi–Harada.

### Pachychoroid-associated diseases

CSC, CNV with increased choroidal thickness, and polypoidal choroidal vasculopathy are all classified as pachychoroid spectrum diseases [43]. When diagnosing CSC, it is therefore important to differentiate it from other diseases on this spectrum, as well as from diseases that can cause serous macular detachment and PED. These include other pachychoroid spectrum disorders, such as peripapillary pachychoroid syndrome, pachychoroid pigment epitheliopathy, focal choroidal excavation and peripheral exudative haemorrhagic chorioretinopathy [44, 45].

### Neovascular diseases

In patients aged over 50 years, CSC should be differentiated from age-related macular degeneration, particularly with type 1 macular neovascularisation [46]. Where soft drusen are present, type 1 CNV in exudative age-related macular degeneration should be suspected. It is difficult to differentiate CSC from exudative age-related macular degeneration in cases of diffuse, ill-defined leakage with unclear boundaries on FA; ICGA can be helpful in such cases. In CSC, multiple hyperfluorescent areas that appear in the early stages decrease later; in contrast, in exudative age-related macular degeneration, hyperfluorescence persists due to staining [41].

ICGA and OCT are also useful in differentiating CSC from polypoidal choroidal vasculopathy [47], with a branching neovascular network and polypoidal lesions being visible in cases of polypoidal choroidal vasculopathy. While the subretinal space is relatively clear in CSC, polypoidal lesions may be observed at the border of PED in polypoidal choroidal vasculopathy, with the branching neovascular network appearing as the double-layer sign [48]. Recently, cases of the double-layer sign characterised by RPE elevations with a greatest transverse linear dimension of 1000 μm or more, an irregular RPE layer with a height predominantly less than 100 μm, and non-homogeneous internal reflectivity have been termed “shallow, irregular RPE elevation”. The presence of shallow, irregular RPE elevation features has been associated with an increased likelihood of macular neovascularisation [49].

### Inflammatory diseases

Early-stage Vogt–Koyanagi–Harada disease is characterised by a prodrome of meningismus and/or flu-like symptoms followed by bilateral diffuse choroiditis, multifocal sensory retinal detachment in the posterior pole, multiple leakage points on FA at the RPE level with or without optic disc inflammation, and mild anterior chamber inflammation. Left untreated, progressive granulomatous anterior uveitis, vitritis and shallowing of the anterior chamber due to swelling of the ciliary body can ensue. However, the disease shows an immediate response to high-dose corticosteroid treatment [50].

Posterior scleritis also causes exudative retinal detachment in the posterior pole [51]. Characteristic findings include thickening of the sclera and “T-sign” as evidenced by B-mode ultrasonography, and deep pain sometimes exacerbated by eye movement. The presence of concomitant anterior scleritis increases the likelihood of posterior scleral inflammation, and severe scleritis can be accompanied by anterior chamber or vitreous inflammatory cells [51].

### Choroidal tumours and systemic malignancies

Choroidal melanoma, choroidal haemangioma, metastasis of malignant tumour, choroidal osteoma and leukaemia infiltration should also be differentiated from CSC [52–54]. Ultrasonography, angiography and computed tomography and/or magnetic resonance imaging are helpful in this respect.

Haematological malignancies such as Waldenström’s macroglobulinaemia, choroidal lymphoma and leukaemia can present with serous retinal detachment resembling CSC [46]. Waldenström’s macroglobulinaemia is characterised by IgM-related hyperviscosity, causing SRF accumulation, while choroidal lymphoma presents with sub-RPE or choroidal infiltration. Leukaemia may also cause bilateral SRF, often related to choroidal infiltrates or RPE dysfunction, with resolution occurring after systemic treatment. Multimodal imaging and systemic evaluations are key to differentiating these conditions from CSC.

Paraneoplastic syndromes, such as bilateral diffuse uveal melanocytic proliferation and paraneoplastic vitelliform maculopathy, can mimic CSC by presenting with serous retinal detachment or vitelliform lesions [46]. Bilateral diffuse uveal melanocytic proliferation is characterised by bilateral pigmented choroidal lesions and rapid cataract progression, often associated with systemic malignancies, while paraneoplastic vitelliform maculopathy features multifocal yellowish subretinal deposits due to autoimmune RPE dysfunction. Differentiation from CSC relies on multimodal imaging, systemic evaluation and electro-oculogram findings.

### Genetic and developmental disorders

Several genetic retinal diseases, such as Best vitelliform macular dystrophy, RP1L1-associated occult macular dystrophy, central areolar choroidal dystrophy and pseudoxanthoma elasticum, can mimic CSC by presenting with serous SRF or similar OCT findings [46]. However, they can be distinguished from CSC based on family history, genetic testing, symmetrical retinal changes, absence of focal leakage on FA, and systemic features (e.g., angioid streaks in pseudoxanthoma elasticum). Understanding these differences is crucial for accurate diagnosis and management.

Ocular developmental anomalies, such as dome-shaped macula, tilted disc with inferior staphyloma, optic disc pit, uveal effusion syndrome, focal choroidal excavation, macular choroidal macrovessel and torpedo maculopathy, can mimic CSC by presenting with SRF [46]. These conditions can be distinguished through OCT features, such as scleral thickening, optic nerve anomalies or choroidal concavities, as well as the absence of focal leakage on FA and differences in systemic associations or clinical progression.

### Drug-induced serous retinopathy

Mitogen-activated protein kinase inhibitor-associated serous retinopathy may show similar features to CSC on OCT; however, it is mainly caused by RPE dysfunction [55]. In addition, SRF accumulation is a well-documented side effect of treatment with fibroblast growth factor receptor inhibitors. In a retrospective analysis of 146 patients receiving fibroblast growth factor receptor inhibitors for the treatment of solid tumours, Francis et al. reported the development of CSC-like retinopathy in 13.7% of the population [56].

## Treatment of CSC

Establishing optimal treatment guidelines for CSC is complicated due to the disease’s natural course and its variety of clinical manifestations. Since most acute CSC cases may recover with no treatment (observation only) within 3–4 months, observation has been considered an appropriate first-line approach. Treatment should be considered if retinal detachment persists for more than 3 months [3, 15].

Considering the relatively good visual prognosis of patients with CSC, the chosen treatment modality must be evidence-based and demonstrate a favourable safety profile, especially since CSC often resolves spontaneously. Most of the studies published to date have analysed retrospective data, and analytical parameters vary in relation to inclusion and exclusion criteria, treatment timing and study outcome. Because CSC often improves or resolves spontaneously, large, prospective, randomised controlled trials conducted over a defined treatment period are of particular interest.

### Reduction of risk factors

As high levels of endogenous or exogenous corticosteroids are associated with the development of CSC, discontinuation of all forms of steroids is recommended [3, 4]. Lifestyle modifications and psychosocial therapy for patients who are prone to psychological stress, and treatment for sleep apnoea where required, are also helpful [15].

### Laser photocoagulation

Navigated laser photocoagulation has been suggested as a safe and effective laser modality for the treatment of CSC associated with extrafoveal leakage on FA [57]. Reported adverse events include CNV at the treatment site [58]. Despite its effectiveness, laser treatment has not been shown to reduce choroidal thickness and therefore may not affect the overall prognosis of CSC. Additionally, no differences in recurrence rate, visual acuity or choroidal thickness have been reported in comparison with observation alone [3]. However, in a non-randomised comparative study of laser treatment versus observation in 45 eyes, Burumcek et al. observed faster fluid resolution, fewer CSC recurrences and better visual acuity after 5 years of follow-up in eyes that were treated with focal laser versus those that received no active treatment [59].

### Sub-threshold laser

To reduce retinal damage from laser photocoagulation while maintaining therapeutic effects, the duration of laser exposure can be decreased and non-visible clinical end points utilised [60]. Several forms of sub-threshold laser are available – namely, sub-threshold micropulse, end-point management and selective. Micropulse diode laser treatment has been widely suggested as an alternative to laser photocoagulation. While conventional lasers can lead to enlargement of final coagulation spots due to thermal damage of the surrounding tissue, the micropulse diode laser has the advantage of minimising unnecessary tissue damage by allowing the tissue to cool before thermal diffusion to the surrounding areas occurs [3].

In a retrospective case series, complete resolution of SRF was noted in 36–100% of patients with chronic CSC after micropulse treatment [61, 62]. Scholz et al. used ICGA-guided 577 nm micropulse laser treatment and found that a second treatment was required in 41% of 42 cases at the 6-week follow-up visit [61]. Other authors investigated several outcomes, including retinal and choroidal thickness, retinal sensitivity and best corrected visual acuity (BCVA), following sub-threshold micropulse laser treatment [63–65]. Several studies reported the favourable outcomes of ICGA-guided 810 nm sub-threshold micropulse laser for chronic CSC. Furthermore, one preliminary study has suggested that ICGA dye may serve to enhance sub-threshold diode laser micropulse photocoagulation for the treatment of chronic CSC, since laser application sites can be verified after treatment using imaging for ICGA but without additional dye injection [66]. However, the effectiveness and usefulness of micropulse laser therapy are controversial, and the lack of an established protocol is a limitation. In the PLACE trial, a subgroup of 79 patients with chronic CSC received high-density sub-threshold micropulse laser treatment; in this subgroup, complete resolution of SRF was reported in 41% and 21% of patients with focal and diffuse leakage, respectively [67].

While sub-threshold micropulse laser treatments are usually targeted at the site of active leakage, end-point management laser is used to treat the entire macular area within a certain range with a standardised pattern [68]. Selective retina therapy, which initially used the protocol of 5-μs argon laser pulses at 514 nm with a repetition rate of 500 Hz, has been shown to selectively destroy the RPE with high peak temperatures around the melanosomes without damaging neurosensory retinal tissue [69]. Although previous studies showed that various sub-threshold laser treatments were effective in reducing SRF and improving the functional outcome, long-term clinical outcomes have yet to be determined [68, 69].

### Photodynamic therapy

Photodynamic therapy (PDT) has been an effective treatment for improving or stabilising visual acuity in patients with CSC. While full-setting PDT has shown significant efficacy in treating CSC, reduced-setting PDT regimens were developed to avoid possible complications, such as profound angiographic closure, which have been reported rarely following PDT for neovascular age-related macular degeneration [70, 71].

Reduced-intensity PDT regimens include half-dose PDT and half-fluence PDT, which are distinct treatment modalities with different photochemical and biological effects. Half-dose PDT refers to a reduction in the amount of verteporfin injected while maintaining full fluence, whereas half-fluence PDT involves halving the fluence after administering the standard dose of verteporfin. These approaches are not interchangeable, as each results in different levels of photochemical activation and therapeutic effects. In PDT, photon energy and the number of active drug molecules do not have a direct compensatory relationship, meaning that adjusting dose and fluence independently leads to distinct biological and clinical outcomes.

The PLACE trial showing the efficacy of half-dose PDT over sub-threshold micropulse laser in CSC demonstrated that changes in choroidal thickness usually decrease after 1 month, often accompanied by resolution of SRF and improvements in visual acuity [3, 72]. A decrease in choroidal thickness suggests that PDT reduces choroidal vascular hyperpermeability, believed to be one of the main pathogenic mechanisms of CSC [73].

In patients with acute CSC, observation or early half-dose or half-fluence PDT with verteporfin may be considered [3, 74]. Both ICGA-guided and FA-guided PDT may be effective in acute CSC [75]. Early PDT is recommended in patients with acute CSC with decreased visual acuity, severe visual discomfort, recurrent episodes (foveal attenuation, cystoid macular degeneration or RPE atrophy) and only one functioning eye, as well as those who choose to receive treatment [75]. Compared with no active treatment, PDT may provide faster resolution of SRF and more rapid recovery of retinal sensitivity [76]. Ozkaya et al. [77] found that 51% of untreated patients with acute CSC had recurrence, compared with 25% of patients treated with low-fluence PDT, while Mohabati et al. [78] reported SRF recurrence in 24% of untreated eyes versus 4% of eyes that received early treatment consisting primarily of FA-guided half-dose PDT. These findings suggest that PDT may decrease the risk of SRF recurrence in patients with acute CSC.

Among the available treatment options, half-dose PDT seems to be the most effective treatment for chronic CSC (Table 3) [3, 79–83]. The long-term efficacy of half-dose PDT is well documented, with SRF resolution rates of 91% [84] and 81% [85] at 19 and 50 months of follow-up, respectively. Moreover, half-dose PDT has been shown to reduce the risk of CSC recurrence compared with observation alone, with rates of 20% versus 53.8% after a minimum follow-up of 3 years [86]. The recurrence rate after half-dose PDT is higher in patients with bilateral versus unilateral chronic CSC [80], and Breukink et al. demonstrated that use of corticosteroids has no impact on outcomes following PDT in patients with chronic CSC [87]. In the PLACE trial, complete resolution of SRF was observed after ICGA-guided half-dose PDT in 51% and 67% of patients with chronic CSC after 6–8 weeks and 7–8 months, respectively, demonstrating superior efficacy over high-density sub-threshold micropulse laser [72]. However, patients with chronic CSC accompanied by extensive foveal RPE atrophy should be counselled regarding the risk of further vision loss following PDT; more studies are needed to clarify this possible adverse effect [3].

**Table 3:** Major studies investigating the efficacy of PDT in CSC.

	Study design	PDT regimen	Patient characteristics	Mean age of patients (years)	Number of eyes	Treatment anatomic outcome (SRF resolution)	Mean follow-up period (months)
Zhao et al. [74]	Randomised clinical trial	50% dose vs 30% dose	Acute CSC	43	131	95% (50% dosing) vs 75% (30% dosing)	12
Fujita et al. [79]	Retrospective interventional case series	Half dose	Chronic CSC	53	204	89%	12
Lai et al. [80]	Retrospective interventional case series	Half dose	Chronic CSC	49	136	97% at 36 months	38
Liu et al. [83]	Retrospective comparative study	Half dose vs half time	Acute CSC and chronic CSC	46	61	91% (half dose) vs 100% (half time)	15
Haga et al. [85]	Retrospective interventional case series	Half dose	Chronic CSC	52	79	94%	50
Sheptulin et al. [81]	Retrospective interventional case series	Half time	Chronic CSC	49	114	87%	12
Roca et al. [82]	Retrospective comparative study	Half dose	Chronic CSC	47	67	95%	17.4
van Dijk et al. (PLACE trial, 2018) [72]	Open-label, randomised clinical trial	Half dose	Chronic CSC	49	89^a^	67%	7

*CSC* central serous chorioretinopathy, *PDT* photodynamic therapy, *SRF* subretinal fluid.

^a^Number of patients.

### Intravitreal injection of anti-VEGF agents

A meta-analysis by Ji et al. failed to confirm the efficacy of bevacizumab, ranibizumab or aflibercept in the treatment of acute CSC; however, the authors did suggest that chronic CSC with type 1 CNV may benefit from anti-vascular endothelial growth factor (VEGF) therapy [88]. Aflibercept is known to have significant effects on choroidal circulation that may facilitate anatomical improvement in chronic CSC. In a prospective, randomised study, three monthly intravitreal aflibercept injections resulted in greater BCVA improvement than sham treatment in patients with CSC of longer than 6 weeks’ duration [89]. Among patients whose disease had lasted for 3 months or more, 50% of eyes in the aflibercept group required additional *pro re nata* (as-needed) injections after 3 months, compared with 100% of eyes receiving sham treatment [89].

The success of some anti-VEGF studies may be due to the inclusion of patients with chronic CSC complicated by subtle type 1 CNV that may be detected by OCT angiography [4]. The standard treatment for CSC complicated by active CNV is intravitreal anti-VEGF injections [46].

### Mineralocorticoid and glucocorticoid receptor antagonists

Some research suggests that eplerenone, a mineralocorticoid receptor antagonist, is effective in the treatment of CSC, and that oral spironolactone is more effective than observation alone, resulting in faster absorption of SRF [90]. However, the randomised, placebo-controlled VICI trial, which evaluated whether eplerenone is superior to placebo in terms of improving BCVA after 12 months of treatment in patients with chronic CSC, failed to meet its primary outcome [91].

Nielsen et al. demonstrated that oral mifepristone, an anti-progestogen, may be effective in the treatment of chronic CSC. The authors found that 44% of patients treated with the drug gained five or more BCVA letters [92]. However, further evidence is needed to fully describe the clinical efficacy of mifepristone in this indication.

### Finasteride

Finasteride, a 5α-reductase inhibitor of dihydrotestosterone, was found to be effective in the treatment of chronic CSC in a small, retrospective study review [93]. However, due to frequent side effects (e.g., loss of libido) and the lack of conclusive evidence of efficacy, finasteride is not currently considered a viable treatment option for CSC.

### Rifampicin

While rifampicin is used primarily for the treatment of tuberculosis and other microbial infections, its effect on cytochrome P450 3A4 induction and on the metabolism of endogenous steroids has led to postulation about its effectiveness as a treatment for CSC. An observational study by Khan et al. found that rifampicin treatment improved BCVA in patients with CSC [94]. However, associated side effects should not be ignored: Nelson et al. reported a case of rifampicin-related hepatotoxicity in a patient with chronic CSC [95].

## Treatments for special subtypes of CSC

### Complex chronic CSC with CNV

The standard treatment for CSC complicated by active type 1 CNV is intravitreal anti-VEGF injections, with several studies demonstrating good efficacy in such cases [46]. The Phase 3 MINERVA study demonstrated that anti-VEGF therapy is more effective than sham injections for CNV of uncommon causes, including in eyes with CNV secondary to CSC [96]. Further research is required to understand the role of PDT for this variation of the disease.

### Multifocal posterior pigment epitheliopathy associated with exudative retinal detachment in CSC (bullous CSC)

Kawamura et al. reported complete resolution of SRF in five of eight patients with atypical CSC (defined as bullous retinal detachment with diffuse or several leakages, severe leakage with fibrin formation under serous retinal detachment, or leakage within a PED) within 1 month of treatment with transpupillary thermotherapy [42]. Since thermotherapy is no longer widely used, PDT might be considered an alternative option for such patients [97]. Additionally, in such treatment-resistant and refractory cases, a combined therapeutic approach may be beneficial, and further discussion on this topic is warranted.

## Discussion and vision academy recommendations for the management of CSC

**Fig. 5:**
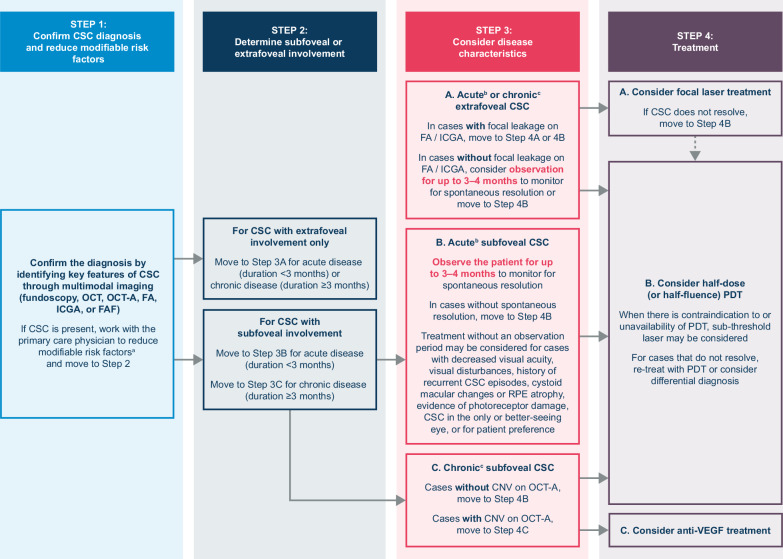
Diagrammatic representation of the proposed treatment guideline for CSC. ^a^Modifiable risk factors may include the use of steroid cream or other drugs, or psychological or physical stress. ^b^Acute CSC is defined as CSC present for <3 months. ^c^Chronic CSC is defined as CSC present for ≥3 months. CNV choroidal neovascularisation, CSC central serous chorioretinopathy, FA fluorescein angiography, FAF fundus fluorescein angiography, ICGA indocyanine green angiography, OCT optical coherence tomography, OCT-A optical coherence tomography angiography, PDT photodynamic therapy, RPE retinal pigment epithelial, VEGF vascular endothelial growth factor.

The classification and treatment of CSC have long been a controversial topic. The most recent conceptualisation of CSC is that it is likely a choroidal disease with chronicity and complications occurring due to increased involvement of the RPE. Several large, multicentre, prospective, randomised controlled trials of CSC treatment have been published in recent years, contributing to the existing evidence base. Knowledge of the updated evidence for the pathogenesis of CSC, as well as risk factors for the disease and treatment outcomes, will provide clinicians with pathophysiology-based treatment guidance. Following a review of the literature, we suggest the following recommendations for the management and treatment of CSC. Figure 5 shows a diagrammatic representation of these recommendations, comprising four key steps:

### Step 1. Confirm the diagnosis of CSC and reduce modifiable risk factors

Confirm the diagnosis by identifying key features of CSC through multimodal imaging. At this stage, it is important to look for the presence of type 1 CNV using OCT angiography and to rule out other diseases that may mimic CSC.

Once the diagnosis of CSC has been confirmed, check for any modifiable risk factors. If present, work with the prescribing or other physicians to reduce modifiable risk factors, such as use of steroid cream or other drugs, or reduction of psychological or physical stress.

### Stage 2. Determine subfoveal or extrafoveal involvement and duration of symptoms

Patients with CSC should be assessed to determine whether there is subfoveal involvement or extrafoveal involvement only. It is also important to establish, wherever possible, whether the disease is acute (duration <3 months) or chronic (duration ≥3 months), as these factors can influence the recommended management options.

### Step 3. Consider disease characteristics

In cases of acute or chronic CSC with extrafoveal involvement only and no obvious focal leakage, consider observation for up to 3–4 months to monitor for spontaneous resolution or commence treatment immediately (see Step 4 below). An observation period of 3–4 months is also recommended for cases of acute subfoveal CSC to monitor for spontaneous resolution. If no improvement is observed initially through serial OCT monitoring, consider treating earlier than 3–4 months, as persistent SRF could lead to vision loss. Early treatment should also be considered in a subset of patients with certain characteristics, namely decreased visual acuity, visual disturbances, history of recurrent CSC episodes, cystoid macular changes or RPE atrophy, evidence of photoreceptor damage, CSC in the only or better-seeing eye, or patient preference for early treatment.

### Step 4. Treatment

In cases of acute or chronic CSC with extrafoveal involvement only, focal laser or half-dose (or half-fluence) PDT should be considered if focal leakage is present on FA or ICGA. In the absence of focal leakage, an observation period of 3–4 months can be considered to monitor for spontaneous resolution. In cases of acute or chronic CSC with subfoveal involvement and absence of type 1 CNV on OCT, half-dose (or half-fluence) PDT is recommended (possibly after an observation period of 3–4 months in acute CSC). Sub-threshold laser treatment may be considered for cases with extensive RPE damage, previous poor response to PDT, contraindication to PDT or unavailability of PDT. In cases of chronic subfoveal CSC with type 1 CNV on OCT angiography, anti-VEGF treatment should be considered.

In conclusion, it is important to determine the baseline clinical characteristics of the patient with CSC in order to determine the optimal timing of treatment as well as the optimal treatment option. Based on efficacy and safety data from retrospective studies and prospective studies such as the PLACE trial, half-dose (or half-fluence) PDT should be considered the treatment of choice for chronic CSC. However, the pathophysiology according to various subtypes and the treatment outcomes of those subtypes still need to be investigated. Future large, multicentre, randomised trials will likely shed more light on the long-term outcomes of PDT and the efficacy of various other treatments, providing a better comparative overview of the principal treatment options that are currently available.
